# Sex is no determinant of cardioprotection by ischemic preconditioning in rats, but ischemic/reperfused tissue mass is for remote ischemic preconditioning

**DOI:** 10.14814/phy2.14146

**Published:** 2019-06-17

**Authors:** Helmut R. Lieder, Amelie Irmert, Markus Kamler, Gerd Heusch, Petra Kleinbongard

**Affiliations:** ^1^ Institute for Pathophysiology West German Heart and Vascular Center Essen University of Essen Medical School Essen Germany; ^2^ Department of Thoracic and Cardiovascular Surgery West German Heart and Vascular Center Essen University of Essen Medical School Essen Germany

**Keywords:** A cute myocardial infarction, cardioprotection, ischemia–reperfusion injury, remote ischemic conditioning

## Abstract

We determined the impact of sex on the magnitude of cardioprotection by local and remote ischemic preconditioning (IPC and RIPC) and of ischemic/reperfused peripheral tissue mass on protection by RIPC. Hearts of female and male Lewis rats were excised, perfused with buffer, and underwent either IPC by 3 × 5/5 min global zero‐flow ischemia/reperfusion (GI/R) or time‐matched perfusion (TP) before 30/120 min GI/R. In a second approach, anesthetized female and male Lewis rats underwent RIPC, 3 × 5/5 min ischemia/reperfusion of one or both hindlimbs (1‐RIPC or 2‐RIPC), or placebo. Thirty minutes after the RIPC/placebo protocol, hearts were excised and subjected to GI/R. In female and male hearts, infarct size was less with IPC than with TP before GI/R (IPC+GI/R_female_: 12 ± 5%; IPC+GI/R_male_: 12 ± 7% vs. TP+GI/R_female_: 33 ± 5%; TP+GI/R_male_: 37 ± 7%, *P* < 0.001). With 2‐RIPC, infarct size was less than with 1‐RIPC in female and male rat hearts, respectively (2‐RIPC+GI/R_female_: 15 ± 5% vs. 1‐RIPC+GI/R_female_: 22 ± 7%, *P* = 0.026 and 2‐RIPC+GI/R_male_: 16 ± 5% vs. 1‐RIPC+GI/R_male_: 22 ± 8%, *P* = 0.016). Infarct size after the placebo protocol and GI/R was not different between female and male hearts (36 ± 8% vs. 34 ± 5%). Sex is no determinant of IPC‐ and RIPC‐induced cardioprotection in isolated Lewis rat hearts. RIPC‐induced cardioprotection is greater with greater mass of ischemic/reperfused peripheral tissue.

## Introduction

The translation of cardioprotection by ischemic conditioning to clinical practice has been largely disappointing so far (Heusch 2017; Davidson et al. 2019a). Such failure of translation is, apart from details of the underlying signal transduction of cardioprotection and strategies of its recruitment (Heusch 2015; Andreadou et al. 2019; Davidson et al. 2019b; Hausenloy et al. 2019a,2019b; Zuurbier et al. 2019), due to the lack of an optimal algorithm of the conditioning stimulus (duration and number of ischemia/reperfusion cycles, as well as its temporal distance to the index ischemia). Clinical Phase II studies to identify an optimal conditioning algorithm do not exist at all. Only few experimental studies have established a dose–response relationship between the conditioning stimulus and the magnitude of infarct size reduction (Skyschally et al. 2009; Johnsen et al. 2016). It is not even clear what exactly defines stimulus strength, to what extent stimulus strength depends on the temporal sequence of ischemia/reperfusion cycles, and for remote ischemic conditioning (RIC) to what extent it depends on the ischemic/reperfused peripheral tissue mass (Heusch 2017). A recent meta‐analysis of animal models on RIC supports no relation between duration or number of ischemia/reperfusion cycles or the mass of ischemic/reperfused peripheral tissue with the magnitude of infarct size reduction (Bromage et al. 2017). In fact, there is only one experimental study in mice with the aim to identify an optimal algorithm of RIC, in which duration and number of ischemia/reperfusion cycles rather than the mass of ischemic/reperfused peripheral tissue determined the magnitude of infarct size reduction (Johnsen et al. 2016). In contrast, in healthy volunteers, there was no difference between RIC on one arm versus one leg in releasing cardioprotective substances into the plasma which then attenuated hypoxia/reoxygenation injury in cultured rat myoblasts (Dezfulian et al. 2017). In patients undergoing mitral valve replacement, however, the combination of arm and leg RIC exerted a stronger cardioprotective effect than RIC on only one arm as reflected by a reduction of biomarker release (Wu et al. 2011).

Sex is a potential confounder of conditioning interventions (Ferdinandy et al. 2014), and the majority of preclinical studies were conducted in males (Bromage et al. 2017). In those few studies comparing cardioprotection between both sexes, local ischemic conditioning reduced infarct size less in female than in male rat hearts (Penna et al. 2009; Ciocci Pardo et al. 2018). Some studies done exclusively in female rats – without males as control – failed to confirm infarct size reduction by local ischemic conditioning and/or RIC (Dow and Kloner 2007; Sachdeva et al. 2014). In contrast, in retrospective analyses of clinical trials on RIC in patients undergoing elective coronary artery bypass grafting (Kleinbongard et al. 2016) or primary percutaneous coronary intervention (Crimi et al. 2013; Eitel et al. 2015; Sloth et al. 2015), there were no differences between the sexes in reduction of biomarker release (Crimi et al. 2013; Kleinbongard et al. 2016) or infarct size on imaging (Eitel et al. 2015; Sloth et al. 2015).

We have now used our established rat model (Lieder et al. 2018) to study the impact of sex on the magnitude of infarct size reduction by local and remote ischemic preconditioning (IPC and RIPC) and the impact of ischemic/reperfused peripheral tissue mass on the magnitude of infarct size reduction by RIPC.

## Material and Methods

Experiments were performed between September 2018 and February 2019. All protocols were approved by the Bioethical Committee of the district of Düsseldorf, Germany (G1413/14, G1625/17, and G1655/18). The experimental protocols, measurements of coronary flow and left ventricular pressure (LVP), quantification of infarct size, and induction of IPC and RIPC were standard (Bøtker et al. 2018; Lindsey et al. 2018). Lewis rats (females: 200–280 g, 14–20 weeks; males: 200–380 g, 10–16 weeks) were obtained from the local animal facility. Female and male rats were randomly assigned to the respective protocols. The estrous cycle in female rats is short (4–5 days) and has no impact on myocardial ischemia/reperfusion injury per se;(Frasier et al. 2013) thus, we did not examine its respective stage.

### Rat hearts ex vivo

**Figure 1:**
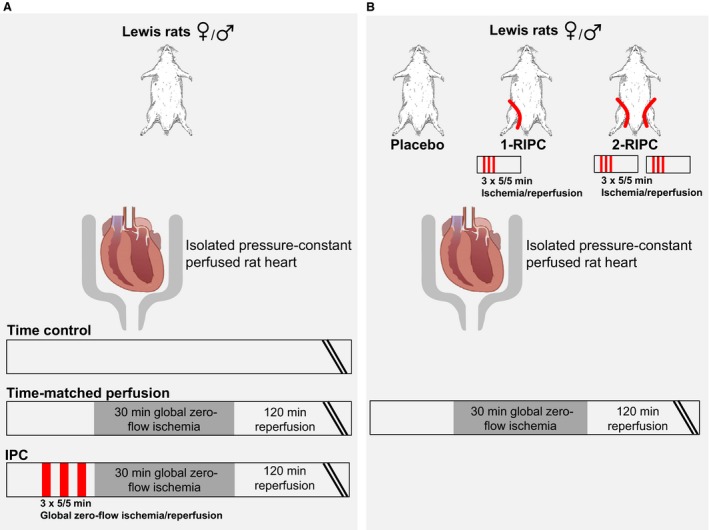
Experimental protocols: (A) Local ischemic preconditioning (IPC); (B) remote ischemic preconditioning (RIPC). I/R: ischemia/reperfusion; 1‐RIPC: one hindlimb RIPC; 2‐RIPC: both hindlimbs RIPC.

The methods were largely identical to those reported in detail before (Lieder et al. 2018). Rats were anesthetized with an intraperitoneal injection of sodium pentobarbital (800 mg/kg; Narcoderm; cp‐pharma, Burgdorf, Germany, supplemented with unfractionated heparin 300 IU/kg). The aorta was cannulated and the heart mounted on a Langendorff apparatus and perfused at constant pressure of 65–70 mmHg with a modified Krebs‐Henseleit buffer (in mmol/L: NaCl 118.0, KCl 4.7, MgSO_4_ 1.6, KH_2_PO_4_ 1.2, glucose 5.6, NaHCO_3_ 24.9, sodium pyruvate 2.0, CaCl_2_ 2.0; gassed with 95% O_2_ and 5% CO_2_ in a prewarmed reservoir). Coronary flow was measured with an inline ultrasonic flowprobe (TS410, Transsonic Systems Inc., Ithaca, NY, USA) above the aortic cannula. A water‐filled latex balloon was inserted into the left ventricular cavity and connected to a pressure transducer (Codan‐PVB, Lensahn, Germany) to measure LVP. End‐diastolic LVP was set to 5–15 mmHg by graded balloon inflation during the initial 5‐min perfusion. Left ventricular developed pressure (LVDP) was calculated as the difference between peak and end‐diastolic LVP. Coronary flow and end‐diastolic and peak LVP were continuously recorded (LabChart 8, AD Instruments Pty LTD, New South Wales, Australia). Hearts were allowed to stabilize for 10–20 min. Preparations with coronary flow <9.0 mL/min (female hearts) or <10.0 mL/min (male hearts) or >18.0 mL/min (female and male hearts) or with LVDP <60 mmHg (female and male hearts) after 10‐ to 20‐min stabilization were excluded. Heart rate was kept constant at 360 beats per min by right atrial pacing. Hearts were immersed in prewarmed oxygenated Krebs‐Henseleit buffer. The temperature of the perfusion and immersion buffers was monitored with probes in the aortic cannula and in the immersion buffer throughout the experiment and kept between 37.2°C and 37.8°C by a heat exchanger next to the aortic cannula. IPC was induced by three cycles of 5 min/5 min global zero‐flow ischemia/reperfusion (GI/R) immediately prior to 30 min/120 min GI/R (IPC+GI/R_female_, *n* = 14; IPC+GI/R_male_, *n* = 15, Fig. 1A). In a control group, time‐matched (for the duration of IPC) 30‐min perfusion (TP) was performed before 30 min/120 min GI/R (TP+GI/R_female_, *n* = 10; TP+GI/R_male_, *n* = 15, Fig. 1A). As a time control, hearts were perfused for a duration equal to that of the other protocols, that is, 200 min without GI/R (time control_female_; time control_male_, *n* = 6 each, Fig. 1A). Coronary flow and LVDP were calculated as mean values during the last minute each of the stabilization period (baseline), at 5‐ and 25‐min ischemia and at 10‐, 30‐, and 60‐min reperfusion, respectively. After the completion of reperfusion, the heart was frozen in Cryomatrix (Thermo Fisher Sientific, Schwerte, Germany) at −20°C and cut into transverse 2‐mm‐thick slices. Infarcted tissue was demarcated by staining with 0.09 mol/L sodium phosphate buffer containing 1.5% triphenyltetrazolium chloride at 37°C for 5 min. Stained slices were photographed from both sides. The total slice area and the infarcted areas were quantified by computer‐assisted planimetry (ImageJ 1.48v, National Institutes of Health, Bethesda, Maryland, USA), and infarct size was calculated as percent of the sum of the left and right ventricular mass (% of ventricular mass).

### Rats in situ

The methods were largely identical to those reported in detail before (Lieder et al. 2018). Lewis rats were anesthetized with an intraperitoneal injection of ketamine/xylazine (100 mg/10 mg per kg). Spontaneously breathing animals received oxygen‐enriched air, were placed on a thermistor‐controlled heating pad, and covered with drapes to prevent hypothermia. The heating pad was adjusted to keep rectal temperature between 36.5°C and 38.0°C. The anesthetic depth was assessed from the pedal withdrawal reflex, respiration, and heart rate. These protocols were performed in situ, whereas myocardial ischemia/reperfusion was induced in isolated rat hearts.

#### RIPC protocol

One‐third of the initial anesthetic drug dosage was again injected intraperitoneally to maintain anesthesia. A tourniquet was placed around one hindlimb (1‐RIPC) or both hindlimbs (2‐RIPC). RIPC was induced by tightening and quick release of the tourniquet(s); dark‐blue skin color was taken to indicate leg ischemia. The position of the tourniquet(s) was marked. Reperfusion was induced by quick release of the tourniquet(s) for 5 min, respectively. The ischemia/reperfusion cycle in the hindlimb(s) was performed three times. Thirty minutes after the last ischemia/reperfusion cycle, unfractionated heparin (300 IU/kg; heparin‐Natrium‐2500‐ratiopharm, Ratiopharm GmbH, Ulm, Germany) was injected intraperitoneally to attenuate coagulation. Thirty minutes after the last ischemia/reperfusion cycle, hearts were excised, perfused, and subjected to 30 min/120 min GI/R, as described above (1‐RIPC+GI/R_female_, *n* = 8; 1‐RIPC+GI/R_male_, *n* = 11; 2‐RIPC+GI/R_female_, *n* = 8; 2‐RIPC+GI/R_male_, *n* = 11, Fig. 1B). Total body mass was weighed. The ischemic/reperfused hindlimb(s) was dissected with respect to the marking(s) on the hindlimb(s), weighed, and expressed as total mass and normalized to total body weight.

#### Placebo protocol

The protocol was identical to that of RIPC, except that tightening of the tourniquet(s) was omitted. Injection of heparin and excision of the heart corresponded to the respective timing in the RIPC group (placebo+GI/R_female_, *n* = 8; placebo+GI/R_male_, *n* = 12, Fig. 1B).

### Statistics

Normality was confirmed for all data sets (Kolmogorov–Smirnov test). Data are presented as means ± SD. Coronary flow and LVDP at baseline, infarct size, body weight, ischemic/reperfused hindlimb mass, and ischemic/reperfused hindlimb mass normalized to body weight were analyzed by two‐way ANOVA (protocol, sex). Coronary flow was different between sexes; therefore, the time courses of coronary flow and LVDP were analyzed within female and male groups separately by two‐way ANOVA for repeated measures. Fisher's least significant difference post hoc tests were used to compare individual mean values when the ANOVA indicated a significant difference. Differences were considered significant at the level of *P* < 0.05 (SigmaStat 3.5, Erkrath, Germany).

## Results

### Local ischemic preconditioning

**Figure 2:**
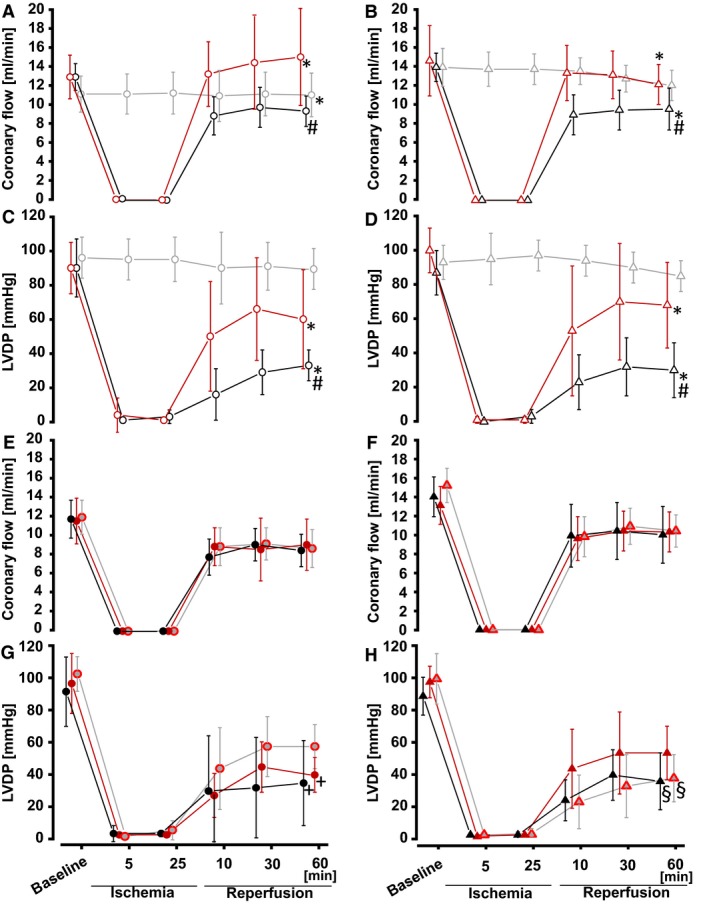
Coronary flow and LVDP in isolated perfused rat hearts. Values are means ± SD. Circles represent female, and triangles represent male rat hearts. (A, B) Coronary flow and (C, D) LVDP in hearts with: local ischemic preconditioning (red: IPC+GI/R), time‐matched perfusion (black: TP+GI/R), time control (grey: time control), (E, F) coronary flow and (G, H) LVDP in hearts with: placebo protocol (black: placebo+GI/R), 1‐RIPC (grey with red margin: 1‐RIPC+GI/R), 2‐RIPC (red: 2‐RIPC+GI/R); *P <* *0.05 versus time‐control, respectively, #*P *<* *0.05 versus IPC+GI/R, respectively +*P < *0.05 versus 1‐RIPC, §*P *<* *0.05 versus 2‐RIPC (two‐way ANOVA).

**Figure 3:**
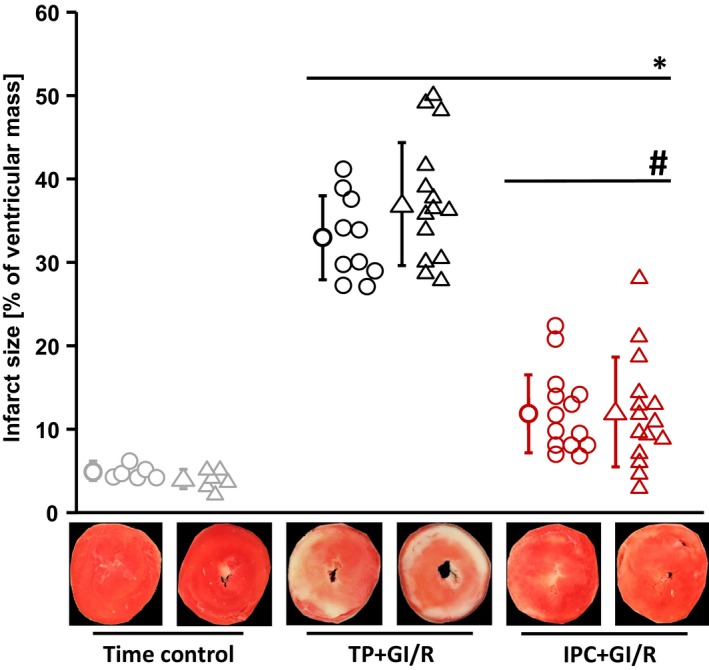
Infarct size in isolated perfused rat hearts with ex vivo local ischemic preconditioning. Closed symbols: means ± SD. Circles represent female rat hearts, and triangles represent male rat hearts. IPC+GI/R: local ischemic preconditioning and 30‐min/120‐min global zero‐flow ischemia/reperfusion; TP+GI/R: time‐matched (for duration of IPC) perfusion and 30‐min/120‐min global zero‐flow ischemia/reperfusion. *P <* *0.001 versus time control, respectively; #*P *<* *0.001 versus TP+GI/R, respectively (two‐way ANOVA and Fisher's least significant difference post hoc tests).

Baseline values for coronary flow were lower in female than in male rat hearts (CF_female_: 12.4 ± 2.2 ml/min vs. CF_male_: 14.2 ± 1.3 ml/min; *P *<* *0.001), but not different within the female and male hearts used in the different protocols (time control; TP+GI/R; IPC+GI/R, respectively, Fig. 2A and B). Baseline values for LVDP were neither different between sexes (LVDP_female_: 92 ± 15 mmHg vs. LVDP_male_: 97 ± 18 mmHg) nor between groups (Fig. 2C and D). The recovery of coronary flow and LVDP during 120‐min reperfusion after 30‐min GI was improved in female and male hearts with IPC (IPC+GI/R_female_ and IPC+GI/R_male_, Fig. 2A–D). With IPC before GI/R, infarct size was less than with time‐matched perfusion before GI/R in both female and male hearts (IPC+GI/R_female_ 12 ± 5%; IPC+GI/R_male_: 12 ± 7% vs. TP+GI/R_female_: 33 ± 5%; TP+GI/R_male_: 37 ± 7%, *P *<* *0.001, Fig. 3). Infarct size was not different between female and male hearts (Fig. 3). Negligible infarction was detected in the respective time control experiments (Fig. 3).

### Remote ischemic preconditioning

Baseline values for coronary flow were lower in female than in male rat hearts (CF_female_: 11.7 ± 2.1 mL/min vs. CF_male_: 14.1 ± 1.9 mL/min; *P *<* *0.001), but not different within the female and male hearts used in the different protocols (placebo+GI/R; 1‐RIPC+GI/R; 2‐RIPC+GI/R, respectively, Fig. 2E and F). Baseline values for LVDP were neither different between sexes (LVDP_female_: 92 ± 19 mmHg vs. LVDP_male_: 93 ± 12 mmHg) nor between groups (Fig. 2G and H). Compared to the placebo protocol, the recovery of LVDP during reperfusion in female hearts was improved with 1‐RIPC, but not with 2‐RIPC (PLA+GI/R_female_; 1‐RIPC_female_; 2‐RIPC_female_; Fig. 2G and H). In male hearts, the recovery of LVDP during reperfusion was improved with 2‐RIPC, but not with 1‐RIPC (PLA+GI/R_male_; 1‐RIPC_male_; 2‐RIPC_male_; Fig. 2G and H). In both sexes, neither 1‐RIPC nor 2‐RIPC improved the recovery of coronary flow compared to placebo protocol (Fig. 2E and F).

**Figure 4:**
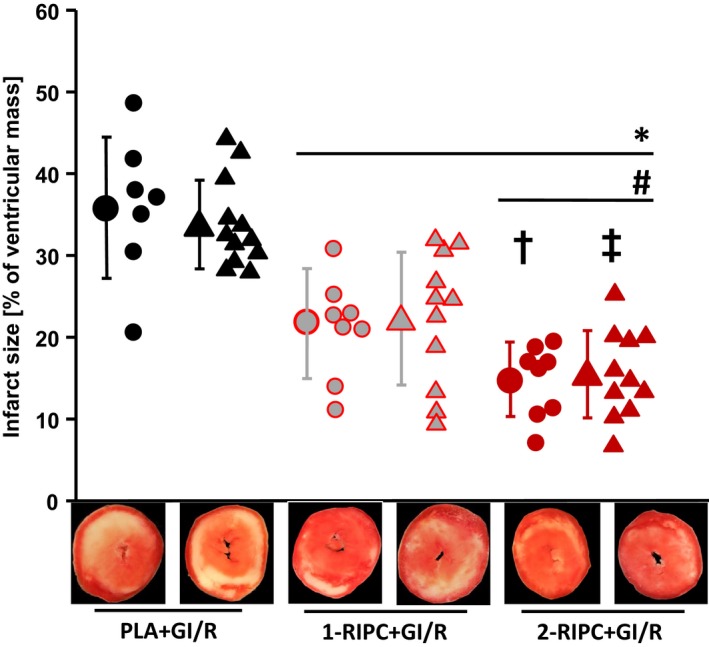
Infarct size in isolated perfused rat hearts with in situ remote ischemic preconditioning. Closed symbols: means ± SD. Circles represent female rat hearts, and triangles represent male rat hearts. Placebo+GI/R: placebo protocol in situ and 30‐min/120‐min global zero‐flow ischemia/reperfusion ex vivo; 1‐RIPC+GI/R: one hindlimb remote ischemic preconditioning in situ and 30‐min/120‐min global zero‐flow ischemia/reperfusion ex vivo; 2‐RIPC+GI/R: both hindlimbs ischemic preconditioning in situ and 30‐min/120‐min global zero‐flow ischemia/reperfusion ex vivo. *P <* *0.001 versus placebo+GI/R, respectively; #*P *<* *0.01 versus 1‐RIPC+GI/R, respectively; †*P *=* *0.026 versus 1‐RIPC+GI/R_female_; ‡*P *=* *0.016 versus 1‐RIPC+GI/Rmale; (two‐way ANOVA and Fisher's least significant difference post hoc tests).

With 1‐RIPC before GI/R, infarct size was less than with placebo protocol in both female and male hearts (1‐RIPC+GI/R_female_: 22 ± 7%; 1‐RIPC+GI/R_male_: 22 ± 8% vs. placebo+GI/R_female_: 36 ± 8%; placebo+GI/R_male_: 34 ± 5%, *P *<* *0.001, Fig. 4). With 2‐RIPC before GI/R, infarct size was further reduced beyond that by 1‐RIPC in both sexes (2‐RIPC+GI/R_female_: 15 ± 5% vs. 1‐RIPC+GI/R_female_, *P *=* *0.026 and 2‐RIPC+GI/R_male_: 16 ± 5% vs. 1‐RIPC+GI/R_male_, *P *=* *0.016, Fig. 4). Ischemic/reperfused hindlimb masses were less in females than in males and larger in 2‐RIPC than in 1‐RIPC, respectively (Table 1). However, ischemic/reperfused hindlimb masses were not different between sexes in 1‐RIPC after normalization to body weight; with 2‐RIPC, ischemic/reperfused hindlimb masses normalized to body weight were higher in female than in male rats (Table 1 *)*.

**Table 1:** Body weight and ischemic/reperfused peripheral tissue mass

Protocol	Body weight [g]	Ischemic/reperfused peripheral tissue mass [g]	Ischemic/reperfused peripheral tissue mass to body weight ratio [% of body weight]
1‐RIPC_female_	205 ± 12*	16 ± 1*	8 ± 0
1‐RIPC_male_	292 ± 24	21 ± 3	7 ± 1
2‐RIPC_female_	216 ± 10*	34 ± 2*#	16 ± 2*#
2‐RIPC_male_	285 ± 22	39 ± 4#	14 ± 1#

1‐RIPC, remote ischemic preconditioning in one hindlimb; 2‐RIPC, remote ischemic conditioning in both hindlimbs.

Values are means ± SD. *P <* *0.05 versus male, respectively; #*P *<* *0.05 versus 1‐RIPC, respectively; two‐way ANOVA with Fisher's least significant difference post hoc tests.

## Discussion

In the present study, infarct size reduction by RIPC was greater with larger mass of ischemic/reperfused peripheral tissue, suggesting a dose–response relationship between stimulus strength and the induced cardioprotection in both sexes. The slight differences in ischemic/reperfused peripheral tissue masses between male and female rats, however, did not impact on RIPC's cardioprotection. We did not observe any sex‐related differences in infarct size, in line with some (Bae and Zhang 2005; Penna et al. 2009; Ciocci Pardo et al. 2018), but not all studies in rats (Li and Kloner 1995; Litwin et al. 1999; Sofia et al. 2014). Rat strain and genetic background (Baker et al. 2000), source of animals (Jones et al. 2015), regional versus global ischemia protocols, their duration, and experimental conditionings (Jones et al. 2015) may have obscured potential sex‐related differences.

We also found no impact of sex on IPC's and RIPC's cardioprotection, in line with the retrospective analyses of clinical trials on RIC in patients undergoing elective coronary artery bypass grafting (Kleinbongard et al. 2016) primary percutaneous coronary intervention (Crimi et al. 2013; Eitel et al. 2015; Sloth et al. 2015). Our results are in some contrast to studies in rats, where the infarct size reduction by local ischemic postconditioning in female hearts was attenuated (Penna et al. 2009; Ciocci Pardo et al. 2018). In these two studies, however, infarct size in females was smaller per se (Penna et al. 2009; Ciocci Pardo et al. 2018), which may have resulted in less cardioprotection along an unknown dose–response relationship.

In line with previous studies (Kaplan et al. 1994; Mitchell et al. 1995), IPC improved the recovery of coronary flow and LVDP during reperfusion. RIPC, however, only impacted on the recovery of LVDP but not on that of coronary flow. IPC may exert stronger protective effects on the coronary vasculature than RIC (Heusch 2016). The recovery of LVDP during reperfusion goes largely along with myocardial infarct size;(Bøtker et al. 2018) however, stunning also contributes to the recovery of LVDP, and the protective effects on infarct size and stunning may differ (Kloner and Jennings 2001; Gelpi et al. 2002).

In conclusion, we have established pragmatically that we can use both female and male hearts for our ongoing studies on the signal transduction of IPC and RIPC. RIPC using ischemia/reperfusion of both hindlimbs reduces infarct size more than using only one hindlimb and improves the recovery of contractile function, emphasizing the existence of a dose–response relationship of cardioprotection. The data in our present study were apparently localized in the flat upper part of such relationship, since the difference between RIPC of one and two hindlimbs was only minor and the greater peripheral muscle mass in males resulted in no greater protection than in females. We realize that the results of our present study may be very specific and limited to our experimental preparations and protocols.

## Conflicts of Interest

The authors declare no conflict of interest.
